# A cross-linguistic evaluation of script-specific effects on fMRI lateralization in late second language readers

**DOI:** 10.3389/fnhum.2014.00249

**Published:** 2014-04-24

**Authors:** Maki S. Koyama, John F. Stein, Catherine J. Stoodley, Peter C. Hansen

**Affiliations:** ^1^Department of physiology, anatomy, and genetics, University of OxfordOxford, UK; ^2^Nathan Kline Institute for Psychiatric ResearchOrangeburg, NY, USA; ^3^Department of Psychology, American UniversityWashington, DC, USA; ^4^School of Psychology, University of BirminghamBirmingham, UK

**Keywords:** visual complexity, orthographic depth, second langauge reading, logogrpahic, functional lateralization

## Abstract

Behavioral and neuroimaging studies have provided evidence that reading is strongly left lateralized, and the degree of this pattern of functional lateralization can be indicative of reading competence. However, it remains unclear whether functional lateralization differs between the first (L1) and second (L2) languages in bilingual L2 readers. This question is particularly important when the particular script, or orthography, learned by the L2 readers is markedly different from their L1 script. In this study, we quantified functional lateralization in brain regions involved in visual word recognition for participants' L1 and L2 scripts, with a particular focus on the effects of L1–L2 script differences in the visual complexity and orthographic depth of the script. Two different groups of late L2 learners participated in an fMRI experiment using a visual one-back matching task: L1 readers of Japanese who learnt to read alphabetic English and L1 readers of English who learnt to read both Japanese syllabic Kana and logographic Kanji. The results showed weaker leftward lateralization in the posterior lateral occipital complex (pLOC) for logographic Kanji compared with syllabic and alphabetic scripts in both L1 and L2 readers of Kanji. When both L1 and L2 scripts were non-logographic, where symbols are mapped onto sounds, functional lateralization did not significantly differ between L1 and L2 scripts in any region, in any group. Our findings indicate that weaker leftward lateralization for logographic reading reflects greater requirement of the right hemisphere for processing visually complex logographic Kanji symbols, irrespective of whether Kanji is the readers' L1 or L2, rather than characterizing additional cognitive efforts of L2 readers. Finally, brain-behavior analysis revealed that functional lateralization for L2 visual word processing predicted L2 reading competency.

## Introduction

The left cerebral hemisphere plays the dominant role in language functioning for most right handed individuals. Differences in functional lateralization can be clinically significant, because weaker leftward lateralization may be an indicator of inefficient or impaired language ability (Chiarello et al., 2012; Bishop, 2013; Gotts et al., 2013). The neuroimaging literature has provided strong evidence for leftward lateralization for language processing (e.g., Xiong et al., 1998; Frost et al., 1999; Tzourio-Mazoyer et al., 2004) including written language processing (e.g., Xue et al., 2004; Seghier and Price, 2011). In particular, the occipito-temporal cortex has been characterized as strongly left lateralized for written words across alphabetic, syllabic, and logographic scripts (Nakamura et al., 2005b; Xue et al., 2006; Seghier and Price, 2011). This contrasts with the longstanding view that the right hemisphere is more specialized for visually complex logographic scripts (e.g., Chinese, Japanese Kanji) (Hasuike et al., 1986 for a review).

Given this strong leftward cortical lateralization as a signature of language processing, an intriguing question arises as to whether the degree of functional lateralization differs between the first language (L1) and second language (L2) in bilinguals, particularly late bilinguals (i.e., late L2 learners) whose L2s are typically less proficient than their L1s. Nelson et al. (2009) addressed this question by examining two types of late L2 reader groups—L2 logographic Chinese readers with alphabetic English as their L1 and L2 English readers with Chinese as their L1. Notably, these two scripts are markedly different in visual complexity, with logographic symbols being visually more complex than alphabetic symbols. Additional activation in the right occipito-temporal cortex was observed only during L2 logographic Chinese reading, but not during L2 alphabetic English reading. This result, indicating weaker leftward functional lateralization for L2 logographic reading in the ventral visual pathway, likely reflects the late L2 readers' extra efforts to cope with the greater visual demands (and thus greater right-hemispheric demand) of processing the L2 logographic symbols. However, considering that logographic reading elicits additional right occipito-temporal activation relative to syllabic reading even in L1 readers (Nakamura et al., 2005a; Koyama et al., 2011), it may be that weaker leftward lateralization for logographic reading in the occipito-temporal cortex reflects the generally increased visual demands when reading logographic symbols, rather than L2 readers' extra efforts to learn the new scripts.

Written languages can also differ in their level of orthographic depth. For example, Japanese Kana has an extremely regular orthography, whereas English has irregular orthography where letters can represent very different sounds in different words. It appears that orthographic depth has little effect on functional lateralization for L2 reading, at least in bilinguals of L1 Spanish (regular orthography) and L2 English (irregular orthography) (Jamal et al., 2012). However, our previous fMRI study indicated that a difference in orthographic depth has a significant impact on L2 functional lateralization in late L2 readers (Koyama et al., 2013). More specifically, stronger leftward lateralization for L2 reading was observed in a phonological region (i.e., the left supramarginal gyrus) only when readers' L2 had a more irregular orthography (i.e., English) than their L1 (i.e., Japanese Kana). This stronger leftward lateralization can be interpreted as L2 readers' extra efforts to cope with the greater phonological demands (and thus greater left-hemispheric demands) during L2 reading (Koyama et al., 2013).

**Figure 1:**
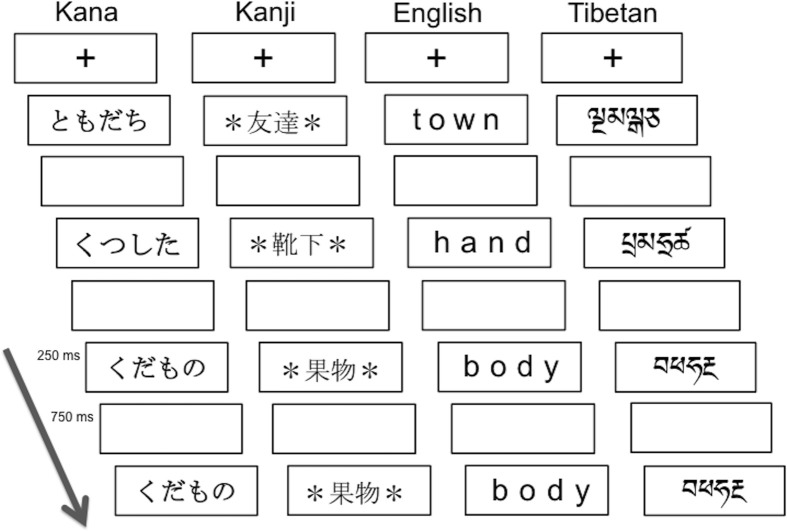
**Schematics of visual one-back matching task**. The paradigm used was a block design with alternating 24-s task blocks and 15-s rest blocks. In each task block, a fixation cross appeared at the center of the visual display, and then 24 words were presented at a rate of 1 per second. The stimulus duration was 250 ms followed by 750 ms blank period, during which participants were asked to press a button when stimuli presented in succession were identical visually. The Kana and Kanji words from the top mean “friend,” “socks,” “fruits,” and “fruits.”

To address questions regarding the effects of visual complexity and orthographic depth on L2 reading cross-linguistically, the current study utilized L1 readers of Japanese, where both syllabic Kana and logographic Kanji are equally used in the writing system, who were also late L2 readers of alphabetic English. We also examined another late L2 group that consisted of L1 readers of alphabetic English who learned to read both Japanese scripts. Visually, Kanji symbols are more complex than Kana and English symbols (Figure 1). Orthographically, English is characterized as having an irregular orthography, whereas Japanese Kana has an extremely regular orthography (Logographic Kanji symbols are primarily mapped onto meanings). Importantly, even with the notable visual differences between Kana and Kanji, a word written in these two Japanese scripts can represent the same sound and meaning, minimizing the influence of confounding factors in language-related experiments.

Such a cross-linguistic evaluation of functional lateralization in two types of late L2 groups can allow us to make the following two predictions: (1) Irrespective of an individual's L1 or L2, logographic reading, relative to non-logographic reading, will be associated with weaker leftward lateralization due to the increased visual demands of processing logographic symbols; and (2) Only in L2 readers of English (whose L1 has a more regular orthography), English reading will be associated with stronger leftward lateralization due to the increased phonological demands. Additionally, we assessed the quantitative relationships between the degree of lateralization and the level of reading ability. Xue et al.'s work (2006) demonstrated that the laterality index of the occipito-temporal cortex while viewing novel symbols predicted subsequent visual word recognition performance for the same symbols. Hence, we hypothesized that functional lateralization in the occipito-temporal cortex would be associated with L2 reading performance, particularly for logographic L2 reading. Although the occipito-temporal cortex is a core region of interest in the current study, we extended our analysis to other brain regions based on the functional maps resulting from our analyses. This approach provides a more comprehensive understanding of the functional lateralization for reading.

## Methods

### Participants

Three groups of skilled adult readers (13 males and 32 females) participated in this study: 1) 15 native Japanese readers who had learnt English as L2 (Japanese-L1/English-L2, the “J1/E2” group; mean age ± *SD* = 29.3 ± 6.4 years), 2) 14 native English readers who had learnt Japanese Kana as L2 (the “E1/J2” group; mean age ± *SD* = 26.2 ± 5.7 years), and 3) 16 native English readers who had no experience of learning either Japanese or logographic Chinese as L2 (“Control” monolingual E1 group; mean age ± *SD* = 25.7 ± 5.3 years). Individuals in the J1/E2 and E1/J2 groups had participated in a previous study (Koyama et al., 2013), and were considered late L2 readers because none of them had early experience (before the age of 12 years) of learning English or Japanese, respectively. In the J1/E2 group, all participants started learning English as L2 at the age of 12 years old, reflecting the official system of English language education in Japan, whereas the mean age of L2-Japanese acquisition in the E1/J2 group was 15.21 (*SD* ± 6.4) years. To estimate which language was the dominant one for the late L2 readers, we asked them to count aloud the number of beans presented to them, in the language with which they felt most comfortable. Counting is usually carried out in the dominant language, in which bilinguals first learned to count (typically L1 in late L2 learners) (Grosjean, 1996). All participants in both the J1/E2 and E1/J2 groups counted in their respective L1s, confirming that their dominant language was indeed the L1. Controls were not fluent in any other language than their L1 (English), confirmed by a questionnaire and a brief interview. All participants were strongly right-handed, as measured by the Annett Handedness Questionnaire (Annett, 1970). They reported no history of psychiatric disorders or learning disabilities (including dyslexia). Participants in the J1/E2 group were either full-time students or exchange students at Universities in the UK, whereas those in the E1/J2 group were full-time students who were studying Japanese at Universities in Oxford. Participants in the Control group were recruited from the University of Oxford and were of similar age and educational level as the L2 groups. The study was approved by the Oxfordshire Research Ethics Committee, and all participants provided written, informed consent to take part in the study.

### Cognitive measures (outside the scanner)

Single word reading competency in English was assessed by the Wide Range Achievement Test III (WRAT; Wilkinson, 1993). For Japanese Kana and Kanji, we used the Kana Word Reading Test (Koyama et al., 2008) and the Kanji Word Reading Test, respectively. For the Kanji Word Reading Test, we selected 60 Kanji words that children are expected to master by the age of 15 in Japan. Unlike the WRAT-III, neither the Kana Word Reading nor Kanji Word Reading tests were standardized, because there was no standardized word reading test in Japanese appropriate for the age range of our participants. Hence, we used the raw scores or percent accuracy for each cognitive test, to perform further statistical analysis. Non-verbal IQ was measured using the Raven's Advanced Progressive Matrices (Raven et al., 1998).

### Tasks performed in the scanner

All participants performed a visual one-back matching task for four types of script: (1) syllabic Kana (Japanese), (2) logographic Kanji (Japanese), (3) alphabetic English, and (4) Tibetan letter-strings, which were visually unfamiliar and unpronounceable to all the participants. Tibetan was chosen as an ecologically valid orthography but with characters equally unfamiliar to all participant groups. Individual Tibetan characters that resembled English or Kana characters were excluded. Figure 1 illustrates the task paradigm and the script conditions. Linguistically, syllabic Kana and alphabetic English are categorized as phonographic scripts where symbols are mapped onto sounds, whereas logographic Kanji symbols can be directly mapped onto meanings. Participants were instructed to press a button with their right index finger as quickly and accurately as possible if successively presented words were visually identical (“Press the button whenever you see two words in succession that are visually identical”).

All the words in the Kana, English, or Tibetan conditions were four characters long, whereas Kanji words were two characters long. As the visual word length of the same word is typically longer when printed in Kana than in Kanji, an asterisk * was placed at the beginning and the end of each Kanji word. This allowed us to equate the retinal image size of the stimuli between word categories. Regarding word frequency, words in all script conditions (with the exception of Tibetan) represented high frequency nouns. Japanese and English words were chosen based on the frequency norms by Amano and Kondo (1999) and those by Kucera and Francis (1967), respectively. Importantly, the Kana and Kanji words were matched in terms of their phonological and semantic features, so that only the visual representations differed from each other. For more details, see Koyama et al. (2011, 2013). The Tibetan letter-strings, which were unfamiliar and unpronounceable stimuli to our participants, were included as a control condition in order to verify that there were no systemic differences in basic visual processing abilities (e.g., recognition, working memory) between the three groups.

The paradigm was a block design with alternating 24 s task blocks and 15 s rest blocks. In the rest block, a small red fixation point was visible at the center of the visual display. In the task block, 24 words were presented at a rate of 1 per second, with an onscreen duration of 250 ms and a blank period of 750 ms between words. Within each task block, 3–5 of the 24 words were visually identical and required a button response. Participants underwent a total of 16 task blocks (4 blocks for each script condition) with 12 rest blocks. Prior to the scan session, participants performed a computerized practice run outside the scanner to ensure task familiarity. In order to prevent word-specific practice effects, the word stimuli used in the practice run were different from the words used in the in-scanner task.

The choice of the visual one-back matching of letter-strings brings both advantages and disadvantages. On the one hand, it allows side-by-side comparison of the different types of orthographic script in all three participant groups studied. On the other hand, the nature of the task is such that it can be performed using purely visual matching without knowledge of the underlying orthography. However, where the orthography has been learned to the high degree needed for efficient reading, we would expect the automaticity of the reading process to be invoked and to reveal language-specific effects. For all the script conditions except for the Tibetan script, each L2 group was likely to yield implicit fMRI activation associated with phonological and/or semantic processes, even in the absence of overt word reading (for alphabetic English, Turkeltaub et al., 2003; for logographic Chinese, Kuo et al., 2004). In the current study, such implicit activation should reflect patterns of functional lateralization for word reading.

### MRI data acquisition

Functional and structural images were acquired with a Varian Siemens 3T scanner at the Center for the Functional Magnetic Resonance Imaging of the Brain in Oxford (FMRIB). Prior to data acquisition, an automated shimming algorithm was applied to reduce magnetic field inhomogeneities (Wilson et al., 2002). For whole brain functional imaging, a T2^*^-weighted gradient-echo EPI sequence was employed with parameters: *TR* = 3000 ms, *TE* = 30 ms, flip angle = 90°, *FOV* = 192 mm^2^, voxel size = 3 × 3 × 3 mm, with 43 slices acquired in axial orientation. The visual one-back matching fMRI protocol consisted of 368 volumes. For structural images, a high-resolution T1-weighted scan was acquired (3D TurboFLASH sequence, *TR* = 13 ms, *TE* = 5 ms, *TI* = 200 ms, flip angle = 8°, *FOV* = 265 mm^2^, voxel size = 1 × 1 × 1 mm).

### MRI data analysis

Data were analyzed using the FMRIB Software Library (FSL, www.fmrib.ox.ac.uk/fsl). The initial four dummy volumes were discarded from the functional MRI data to eliminate non-equilibrium effects of magnetization. The following pre-processing procedures were applied: a high-pass filter cut-off of 40 s, motion correction using MCFLIRT, regular-up slice-timing correction, and spatial smoothing using a Gaussian spatial filter with kernel size 5 mm full width half maximum. The registration of functional images for each participant into standard space was carried out using the FMRIB Non-Linear Image Registration Tool (FNIRT).

After the pre-processing, statistical analysis at the individual level was performed for all the conditions using a general linear model (GLM) with local autocorrelation correction (FILM prewhitening; Woolrich et al., 2001). At the single subject level, contrast images were generated for all participants for each word condition vs. rest (baseline). Rather than using the unpronounceable Tibetan condition as the baseline condition, the rest period was used instead, for the following reasons. Firstly, showing the degree (or lack thereof) of functional lateralization for the Tibetan condition, which can be processed only visually, allows us to highlight the pattern of functional lateralization associated with language processing involved in pronounceable script conditions (see Seghier and Price, 2012). Secondly, the selection of the baseline condition differentially affects observed brain activation (Newman et al., 2001), and thus the use of the Tibetan condition, which dominantly involves visual processing, as a baseline (i.e., subtracting low-level visual processing) can be problematic in delineating brain activation for our other script conditions, among which the level of visual processing demand is likely to differ.

To help correct for motion-related artifacts, the six motion correction parameters estimated with MCFLIRT were included in the model as regressors of no interest. Script conditions were modeled using a Gaussian hemodynamic response function. In addition, in order for the model to best fit the time course of the actual data acquisition, temporal derivatives of the main conditions were added as separate regressors and temporal filtering was applied. Group analysis was performed with random effects analysis using FLAME. Gaussian Random Field theory was used for thresholding (voxel-level *Z* > 2.3, cluster-level *p* < 0.05, corrected for multiple comparisons). This group analysis produced resultant *Z*-value activation maps for each script condition for each group.

#### Regions of interest (ROIs)

**Figure 2:**
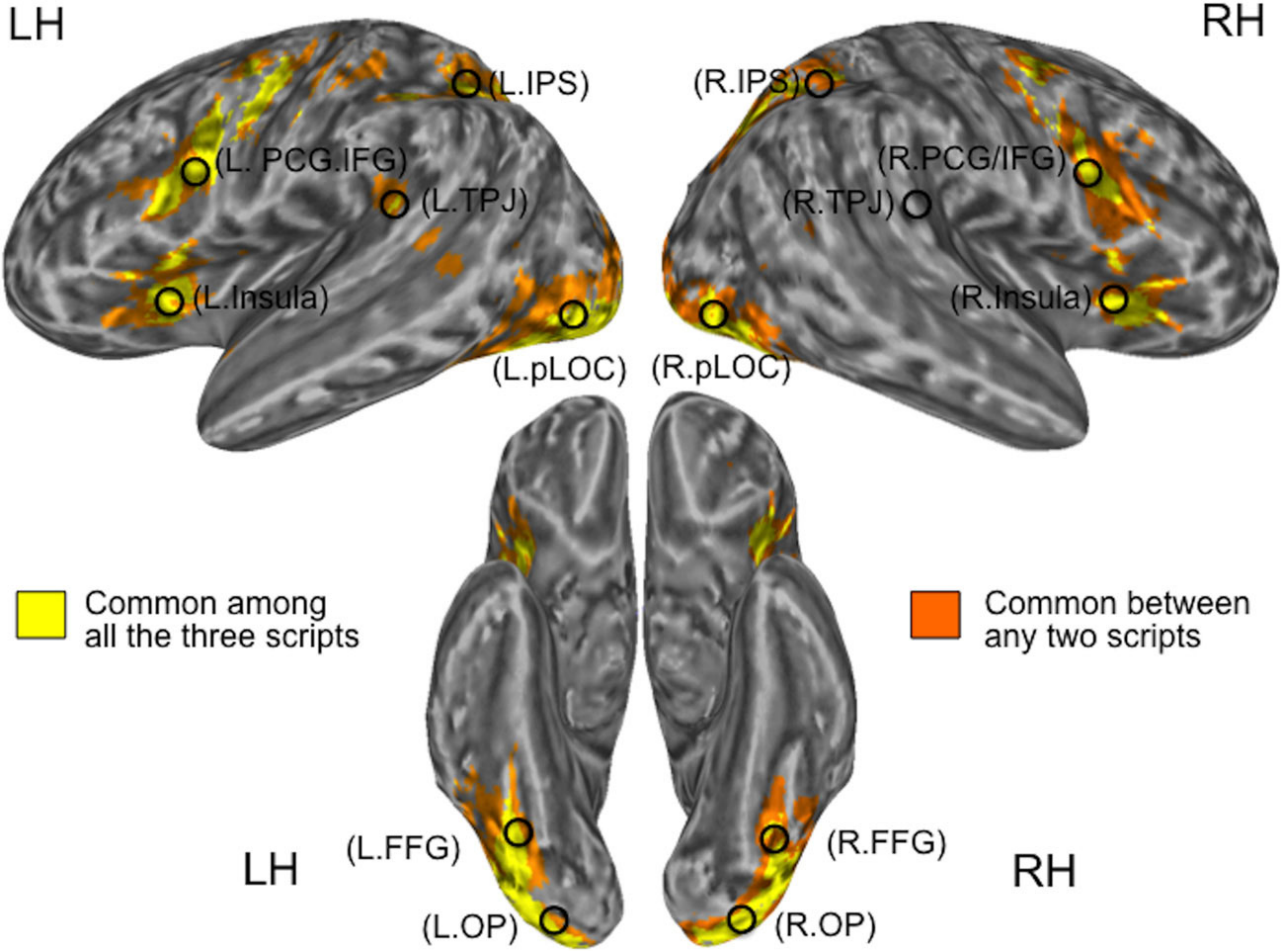
**Locations of seed regions**. Clusters in yellow represent common activation among the three pronounceable script conditions (Kanji, Kana, and English), whereas those in orange represent common activation between any two pronounceable script conditions. Activation patterns for the script conditions were derived from the respective L1 groups. L, Left; R, Right; OP, Occipital Pole; pLOC, posterior Lateral Occipital Complex; TPJ, Temporo-Parietal Junction; IPS, IntraParietal Sulcus; PCG/IFG, PreCentral Gyrus/Inferior Frontal Gyrus.

To investigate functional lateralization in regions involved in word reading, we created regions of interest (ROIs) based on the functional activation patterns during the visual one-back matching task. To create the ROIs, first we combined the three group-level activation maps thresholded at *p* < 0.05 (corrected) for three pronounceable script conditions—Kana, Kanji, and English—those obtained from the respective L1 groups (i.e., the Kana and Kanji conditions from the J1/E2 group; the English condition from the E1/J2 group). The resultant combination map for group-level activation was then binarized to obtain clusters of overlap that contained voxels activated by the three script conditions (Figure 2). Consequently, we identified 11 clusters (6 in the left hemisphere and 5 in the right hemisphere) as being activated by all 3 conditions: the bilateral occipital pole (OP), bilateral posterior lateral occipital complex (pLOC), bilateral intraparietal sulcus (IPS), bilateral precentral gyrus/inferior frontal gyrus (PCG/IFG), bilateral insula, and left temporo-parietal junction (TPJ).

Based on the well-demonstrated word-specific activation in the left fusiform gyrus (FFG), which is known as the Visual Word Form Area (Cohen et al., 2002; Dehaene et al., 2002), we also examined two FFG sub-clusters which were located within the pLOC clusters. For each cluster (and sub-cluster), we created a 6 mm-radius spherical seed centered on the peak MNI coordinates (note: in order to detect the peak MNI coordinates, the combination map for the group-level activation was masked by the binary map and then averaged across the time-series). In addition, we created a sphere centered on the right hemisphere homolog of the left tempo-parietal junction (note; the right temporo-parielta junction was not commonly activated by the three script conditions). These procedures resulted in the creation of 14 seeds for 7 regions of interest (Table 1 and Figure 2).

**Table 1:** **MNI coordinates of seeds**.

**Seeds**	**Left hemisphere**				**Right hemisphere**			
	***x***	***y***	***z***	***z*-score**	***x***	***y***	***z***	***z*-score**
OP	−24	−98	−6	4.36	26	−98	−6	4.90
pLOC	−40	−78	−10	5.07	46	−70	−8	3.55
FFG^*^	−44	−58	−20	3.32	40	−64	−20	3.17
TPJ	−50	−40	22	5.20	50	−40	22	NA
IPS	−24	−66	46	4.28	32	−56	44	4.44
PCG/IFG	−42	4	26	5.43	46	4	28	3.58
Insula	−30	20	−4	4.28	28	24	−4	5.83

ROIs, Regions of Interest; OP, Occipital Pole; pLOC, posterior Lateral Occipital Complex; FFG, Fusiform Gyrus; TPJ, Temporo-Parietal Junction; IPS, IntraParietal Sulcus; PCG/IFG, PreCentral Gyrus/Inferior Frontal Gyrus. ^*^Derived from a sub-cluster within the pLOC cluster.

To confirm that two seeds, which appeared to be homologs in the left and right hemispheres, were actually located in homologous regions, we first visually inspected the clusters superimposed on the Harvard-Oxford Cortical Structural Atlas. In addition, we then calculated the Euclidean distance between pairs of homologous seeds from the peak MNI coordinates, having mirrored the left hemisphere coordinates into the right hemisphere. For example, with the pLOC seeds, we computed the separation between MNI coordinates (Left pLOC: −46, −70, −8) and (Right pLOC: 40, −78, −10), where the x-coordinates were mirror flipped about the x-axis (to yield 46, −70, −8 for the left pLOC). No pair of homologous seeds had a peak separation greater than 13 mm (2 mm for OP, 10.2 for pLOC, 7.2 for FFG, 0 for TPJ, 12.9 for IPS, 4.5 for PCG/IFG, and 4.7 for the insula). These results verify that all seed ROIs, except for the temporo-parietal junction, exhibited significant bilateral activation for all the word conditions.

To obtain the BOLD signal changes in each seed, we extracted the contrast of parameter estimates (COPE) values using FSL Featquery for each script condition for each individual participant. The maximum value within the respective seed was taken, rather than the mean value, to avoid the misinterpretation of resultant laterality index values that can be caused by negative BOLD signal changes (Jansen et al., 2006; Seghier, 2008 for review). Although it is relatively common to use mean values for ROI analyses, recent studies have indicated that the maximum and 90th percentile measures of percent BOLD signal change can be considered to be more representative measures of a typically active voxel within the ROI (Arthurs and Boniface, 2003; Buck et al., 2008).

### Laterality index (LI)

For each ROI, a laterality index (LI) was calculated based on the magnitude of BOLD signal changes for each script condition for each individual. In general, the degree of activation is considered to be a more robust LI measure than the number of activated voxels (Jansen et al., 2006). We used the formula: LI = (RH − LH)/(RH + LH), where LH and RH represent the percent signal change for the left and right hemispheres, respectively. A negative LI value indicates a tendency to leftward lateralization, whereas a positive LI value indicates rightward lateralization. LI values range from −1 (only active in the LH) to +1 (only active in the RH), and participants with LI < −0.2 were considered as having left hemisphere dominance, and those with LI > 0.2 were categorized as having right hemisphere dominance (see Seghier, 2008 for review). Differences between the L1 and L2 script conditions within each group, as well as those between groups for each condition, were tested using pair-wise and independent t statistics, respectively.

## Results

### Cognitive performance outside the scanner

Table 2 gives a summary of the demographic and cognitive measures (performed outside the scanner) for each group. Although all participants achieved high performance on English word reading (WRAT), the three groups differed in their WRAT accuracy scores (*F* = 8.0, *p* < 0.01); the mean accuracy was significantly lower in the English L2 group (J1/E2) than in the two native English groups (for the E1/J2 group *t* = 3.2, *p* < 0.01; for the control group *t* = 3.2, *p* < 0.01). For the Kana Word Reading Test, all participants in the Japanese L1 group (J1/E2) achieved 100% accuracy, and there was a ceiling effect (91% accuracy) in the L2 group of Japanese (E1/J2). The ceiling effect observed in the E1/J2 group is in line with a previous finding that even pre-school Japanese children are able to achieve extremely high accuracy in regular Kana word reading (Shimamura and Mikami, 1994). However, the mean reaction time for Kana word reading differed significantly between the two groups: the J1/E2 group read more quickly than the E1/J2 group (*t* = 5.3, *p* < 0.001). Hence, for further analyses, we used only the reaction time for Kana reading. For the Kanji Word Reading Test, mean accuracy differed significantly between the groups, with the J1/E2 group scoring significantly higher than the E1/J2 group (*t* = 18.0, *p* < 0.001). These results demonstrate that L2 reading competency had not reached native levels in either L2 group.

**Table 2:** **Demographic and cognitive profiles for each group**.

	**J1/E2**	**E1/J2**	**Control**	**Group Diff**.
	**Mean (*SD*)**	**Mean (*SD*)**	**Mean (*SD*)**	
Age	29.3 (6.4)	26.2 (5.7)	25.7 (5.3)	N.S.
Gender	4M/11F	4M/10F	5M/11F	N.S.
Raven	29.1 (5.8)	27.3 (4.6)	28.5 (7.2)	N.S.
WRAT-AC	31.5 (5.5)	37.0 (3.6)	38.4 (3.5)	*P* < 0.01
Kana-AC	20.0 (0.0)	18.1 (1.3)	N.A.	N.A.
Kana-RT	35.6 (7.9)	67.4 (22.0)	N.A.	*P* < 0.01
Kanji-AC	57.3 (2.6)	22.2 (7.0)	N.A.	*P* < 0.01

*J1/E2, Japanese L1/English L2 group; E1/J2, English L1/Japanese L2 group; Diff., Difference; SD, Standard Deviation; M, Male; F, Female; Raven, the Raven's Advanced Progressive Matrices (max. = 36); WRAT, the Wide Range Achievement Test (max. = 44); AC, Accuracy (number correct out of the respective maximum scores); RT, Response Time; Kana, Kana Word Reading Test (max. = 20); Kanji, Kanji Word Reading Test (max. = 60)*.

### In-scanner task performance accuracy

Table 3 summarizes the mean accuracy scores for each script condition in each group. Among the scripts, accuracy was higher for the L1 condition(s) than the L2 condition(s) in each L2 group: the J1/E2 group exhibited significantly higher performance for the two L1 conditions relative to the L2 English condition (relative to Kana, *t* = 5.6, *p* < 0.01; relative to Kanji, *t* = 6.0, *p* < 0.01). Similarly, the E1/J2 group exhibited significantly higher performance for the L1 English condition than the two L2 conditions (relative to Kana, *t* = 4.1, *p* < 0.01; relative to Kanji, *t* = 5.4, *p* < 0.01). As expected, the control group exhibited significantly higher accuracy for the L1 English condition relative to the other conditions (Kana, Kanji, Tibetan) that were unfamiliar and unpronounceable to this group.

**Table 3:** **In-scanner task performance accuracy (% correct)**.

	**J1/E2**	**E1/J2**	**Control**	**Group Diff**.
	**Mean (*SD*)**	**Mean (*SD*)**	**Mean (*SD*)**	
Kana %	96 (0.7)	92 (3.3)	88 (10.2)	*p* < 0.01
Kanji %	93 (1.2)	88 (5.7)	81 (12.7)	*p* < 0.001
English %	91 (1.2)	97 (1.0)	99 (1.7)	*p* < 0.001
Tibetan %	86 (10.4)	87 (9.6)	88 (7.7)	NS

*J1/E2, Japanese L1/English L2 group; E1/J2, English L1/Japanese L2 group; Diff., Difference; SD, Standard Deviation; NS, not significant*.

Among the groups, the mean accuracy differed in the Kana, Kanji and English conditions. For the Kana condition, the J1/E2 group exhibited significantly higher accuracy than the control group (*t* = 3.0, *p* < 0.01), but not the E1/J2 group. For the Kanji condition, the J1/E2 group exhibited significantly higher accuracy than both the E1/J2 (*t* = 3.3, *p* < 0.01) and control groups (*t* = 3.3, *p* < 0.01). For the English condition, the mean accuracy was significantly lower in the J1/E2 group than in the two groups of L1 English readers (the E1/J2, *t* = 4.6, *p* < 0.01; the control group, *t* = 10.1, *p* < 0.001). Notably, for the Tibetan condition, the three groups did not differ in mean accuracy scores (*F* = 0.18, *p* = 0.83). This indicates that the three groups were well-matched for basic visual processing abilities.

### Laterality index for each ROI

**Figure 3:**
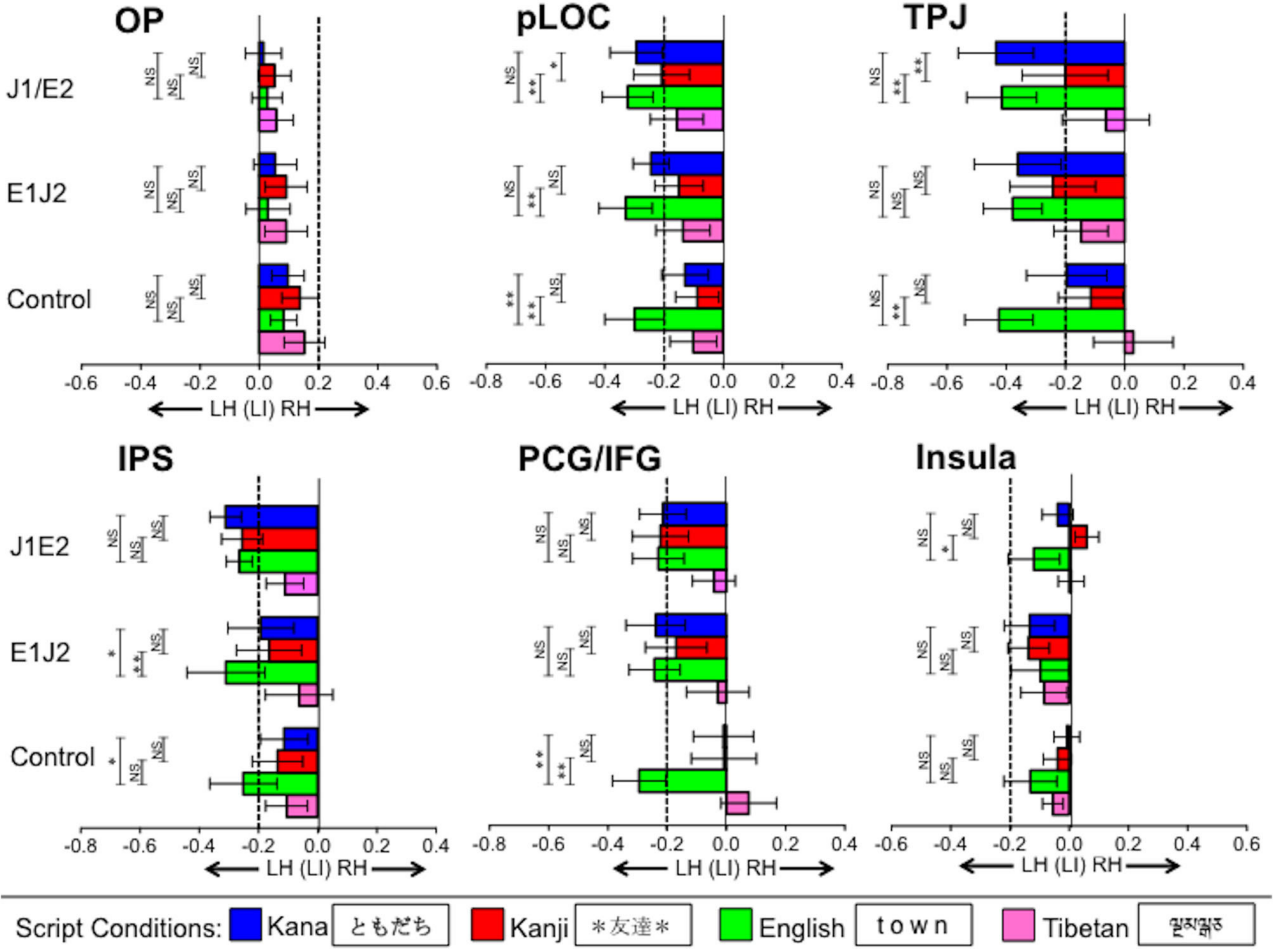
**Functional lateralization in regions of interest (ROIs) for each script condition**. Bars going beyond the dotted line on the left (more negative than −0.2) and right (more positive than 0.2) indicate strong leftward and rightward lateralization, respectively. Vertical error bars on data points represent the standard error of the mean. LH, Left Hemisphere; RH, Right Hemisphere; OP, Occipital Pole; pLOC, posterior Lateral Occipital Complex; TPJ, Temporo-Parietal Junction; IPS, IntraParietal Sulcus; PCG/IFG, PreCentral Gyrus/Inferior Frontal Gyrus; J1/E2, Japanese L1/English L2 group; E1/J2, English L1/Japanese L2 group; Control, Monolingual English L1 control group; ^**^p < 0.01, ^*^p < 0.05.

Before addressing our two primary questions, we report the patterns of functional lateralization for each of the L1 and L2 conditions in each group. The mean laterality index values are illustrated in Figure 3 for the primary 6 ROIs and in Supplementary Figure 1A. for the FFG. Laterality index values smaller than −0.2 and values larger than 0.2 are considered to be significantly left-lateralized and right-lateralized, respectively (Seghier, 2008). In all the groups, significant leftward lateralization was observed for their respective L1s in pLOC, TPJ, IPS, PCG/IFG, and FFG (but not in either OP or insula), replicating previous findings showing strong leftward lateralization for L1 word reading within the known reading network (e.g., Xue et al., 2004; Seghier and Price, 2011).

For the respective L2s, in the J1/E2 group, significant leftward lateralization for L2 English was observed in the majority of the ROIs, except for OP and insula. Similarly, in the E1/J2 group, significant leftward lateralization for L2 Kana was observed in the majority of the ROIs, except for the OP and insula, whereas L2 Kanji exhibited significant leftward lateralization only in the TPJ. Neither L2 group showed leftward lateralization in the FFG (the sub-ROI within the pLOC) for the L2 conditions.

For the Tibetan condition, during which activation was expected to be associated only with visual processing (e.g., recognition, working memory), no group exhibited significant leftward lateralization in any ROI. Of note, this unpronounceable letter-string condition exhibited weak (though not statistically significant) leftward lateralization in the IPS and pLOC, the latter of which is consistent with a previous study (Seghier and Price, 2011). In the control group, as expected, significant leftward lateralization was observed only for the English condition. These results verify that activation during this fMRI task can serve to assess the functional lateralization of word reading.

#### Within-group comparisons

Below, we describe the patterns of functional lateralization for each script condition in each group. We performed pair-wise *t*-tests to compare the L2 script conditions to the L1 script conditions for each ROI in each L2 group (J1/E2 and E1/J2).

***Occipital pole (OP)***. This region exhibited no significant lateralization in any script condition in any group. No significant differences were observed between L1 and L2 conditions in either the J1/E2 group or the E1/J2 group.

***Posterior lateral occipital complex (pLOC)***. In the J1/E2 group, no difference in functional lateralization was observed between the L1 Kana and L2 English conditions (*t* = 0.96, *p* = 0.35), but the pLOC activation was significantly more left lateralized for the L2 English condition relative to the L1 Kanji condition (*t* = 3.07, *p* < 0.01). In the E1/J2 group, pLOC was significantly more left lateralized for the L1 English condition relative to the L2 Kanji condition (*t* = 3.07, *p* < 0.01), but not for L1 English relative to the L2 Kana condition (*t* = 1.25, *p* = 0.23). When looking at differences between the two Japanese scripts (Kana vs. Kanji), in the J1/E2 group the leftward lateralization of pLOC was significantly stronger for the L1 Kana condition than the L1 Kanji condition (*t* = 2.81, *p* < 0.05). Though not statistically significant (*t* = 1.23, *p* = 0.24), the E1/J2 group also exhibited a tendency toward stronger leftward lateralization for the L2 Kana condition than the L2 Kanji condition in this region. That is, irrespective of whether it was participants' L1 or L2, the logographic Kanji condition tended to show weaker leftward lateralization relative to the syllabic Kana and alphabetic English conditions.

***Fusiforum gyrus (FFG)***. In the J1/E2 group, no difference in functional lateralization was observed either between the L1 Kana and L2 English conditions or between the L1 Kanji and L2 English conditions. In the E1/J2 group, the FFG activation was significantly more left lateralized for the L1 English condition relative to the L2 Kanji condition (*t* = 2.3, *p* < 0.05).

***Temporo-parietal junction (TPJ)***. This region's lateralization patterns were similar to those observed in pLOC. In the J1/E2 group, the TPJ was significantly more left lateralized for the L2 English condition relative to the L1 Kanji condition (*t* = 3.05, *p* < 0.01), but no difference was observed between the L1 Kana and L2 English conditions (*t* = 0.19, *p* = 0.85). In the E1/J2 group, there was no significant difference between the L1 English and L2 Kanji conditions (*t* = 0.83, *p* = 0.41). No significant difference was observed between the L1 English and L2 Kana conditions (*t* = 0.10, *p* = 0.92). When comparing the two Japanese scripts, in the J1/E2 group leftward lateralization of the TPJ was significantly stronger for the L1 Kana condition than the L1 Kanji condition (*t* = 3.39, *p* < 0.01). Though not statistically significant (*t* = 0.51, *p* = 0.62), the E1/J2 group exhibited a tendency toward stronger leftward lateralization for the L2 Kana condition than the L2 Kanji condition. Similar to the pLOC, irrespective of whether it was L1 or L2, the logographic Kanji condition tended to show weaker leftward lateralization in the TPJ relative to the syllabic Kana and alphabetic English conditions.

***Intraparietal sulcus (IPS)***. In the J1/E2 group, no significant group difference in laterality index was observed between L1 Kana or Kanji and the L2 English script. However, in the E1/J2 group, the IPS was significantly more left lateralized for the L1 English condition compared to both the L2 Kanji script (*t* = 3.08, *p* < 0.01) and the L2 Kana script (*t* = 2.29, *p* < 0.05). No group showed significant differences between the Kana and Kanji conditions in the IPS.

***Precentral/Inferior frontal gyrus (PCG/IFG)***. In the J1/E2 group, there were no significant differences between either of the L1 conditions and the L2 English condition (Kana vs. English: *t* = 0.41, *p* = 0.69; Kanji vs. English: *t* = 0.19, *p* = 0.85). Similarly, no L1 vs. L2 differences were observed in the E1/J2 group (English vs. Kana: *t* = 0.04, *p* = 0.97; English vs. Kanji: *t* = 0.97, *p* = 0.35). No group showed a significant difference between the Kana and Kanji conditions.

***Insula***. Similar to the OP, this region exhibited no significant lateralization in any script condition in any group. When L1 and L2 conditions were compared, in the J1/E2 group the L2 English condition was significantly more left lateralized relative to the L1 Kanji condition (*t* = 2.28, *p* < 0.05), but not relative to the L1 Kana condition (*t* = 0.99, *p* = 0.34). The E1/J2 group showed no significant differences between the L1 English condition and the L2 Japanese conditions in the insula.

#### Confirmatory analysis between groups

**Figure 4:**
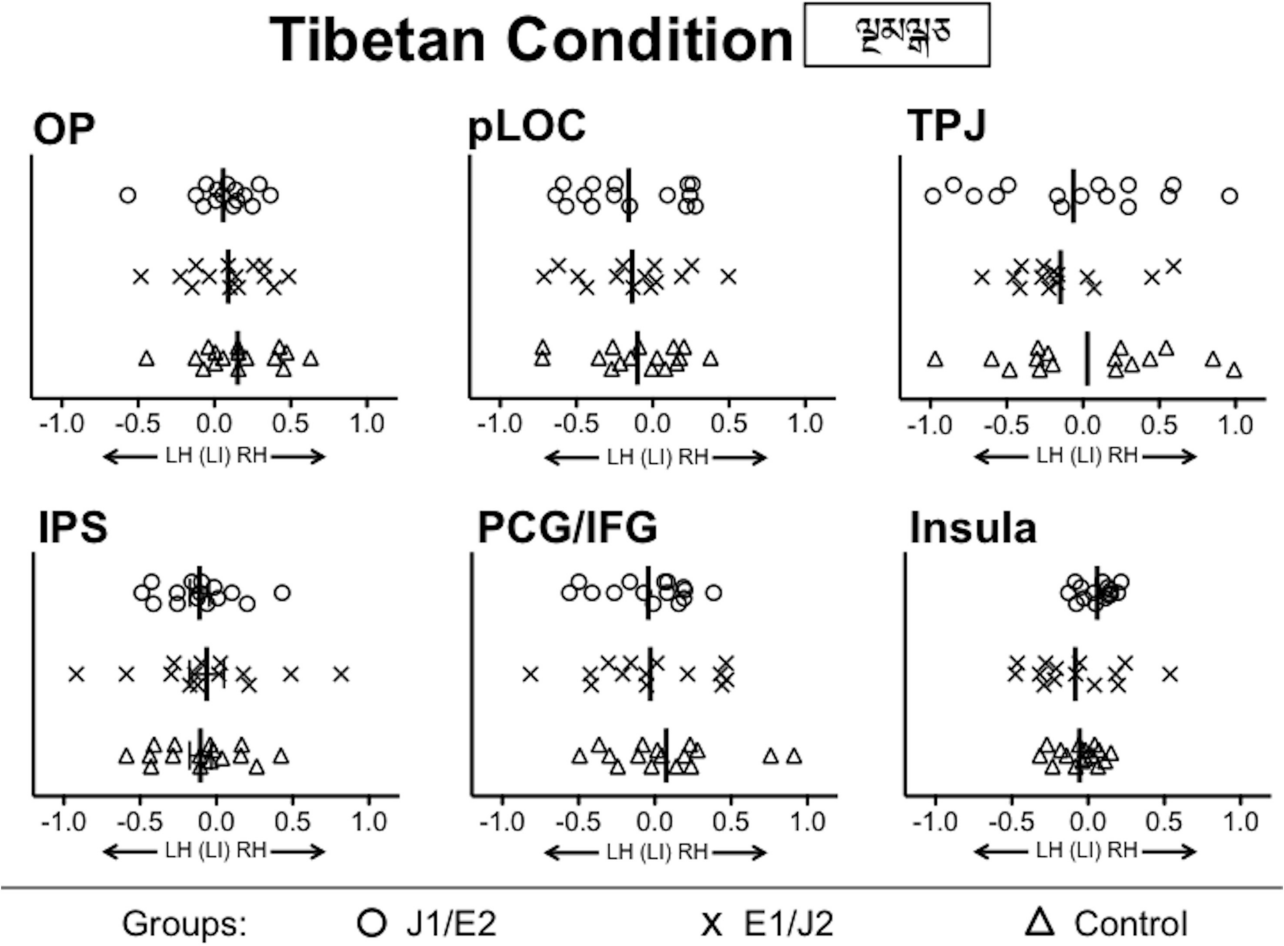
**Group comparisons of functional lateralization for the Tibetan condition**. No significant group difference was observed in any of ROIs. LH, Left Hemisphere; RH, Right Hemisphere; OP, Occipital Pole; pLOC, posterior Lateral Occipital Complex; TPJ, Temporo-Parietal Junction; IPS, IntraParietal Sulcus; PCG/IFG, PreCentral Gyrus/Inferior Frontal Gyrus; J1/E2, Japanese L1/English L2 group; E1/J2, English L1/Japanese L2 group; Control, Monolingual English L1 control group.

First, there was no group difference for the Tibetan condition in any ROI (Figure 4 and Supplementary Figure 1B), confirming that all the groups were well-matched on basic visual processing abilities. This result excludes the possibility that group differences in basic visual processing skills might have confounded patterns of functional lateralization for the pronounceable word conditions during the visual one-back matching task. Second, for the English condition—which all groups were able to read—there were no group differences in the degree of leftward lateralization in any ROI. For each of the Japanese script conditions, no significant difference between L1 and L2 groups was observed in any ROI except for the insula. These results from the group comparisons suggest that the degree of leftward lateralization for word reading was not always stronger for L1 than L2 readers, even when reading proficiency was higher in L1 readers than in L2 readers.

### Confirmatory analysis in pLOC

Seghier and Price (2011) emphasize that a pattern of functional lateralization in the ventral part of the occipito-temporal cortex needs to be considered in lights of the relative contribution of right hemisphere activation. Hence, we plotted BOLD signal changes in the left and right hemisphere of pLOC for each script condition relative to the resting in the J1/E2 group (Supplementary Figure 2). For all the conditions except for the Tibetan condition, the magnitude of BOLD signal changes was significantly higher in the left hemisphere than the right hemisphere, leading to significant leftward lateralization for the pronounceable script conditions. However, the Kanji condition yielded greater activation in the right pLOC relative to the Kana and English conditions (*F* = 8.8, *p* < 0.01), but this pattern was absent in the left pLOC. This relatively stronger right pLOC activation contributed to the observed weaker leftward lateralization in pLOC for the logographic Kanji condition. Our result is consistent with a previous observation that right hemisphere activation is disengaged from the left hemisphere activation in this region specifically for processing words (but not for unfamiliar letter-strings) (Seghier and Price, 2011).

#### Behavioral relevance

**Figure 5:**
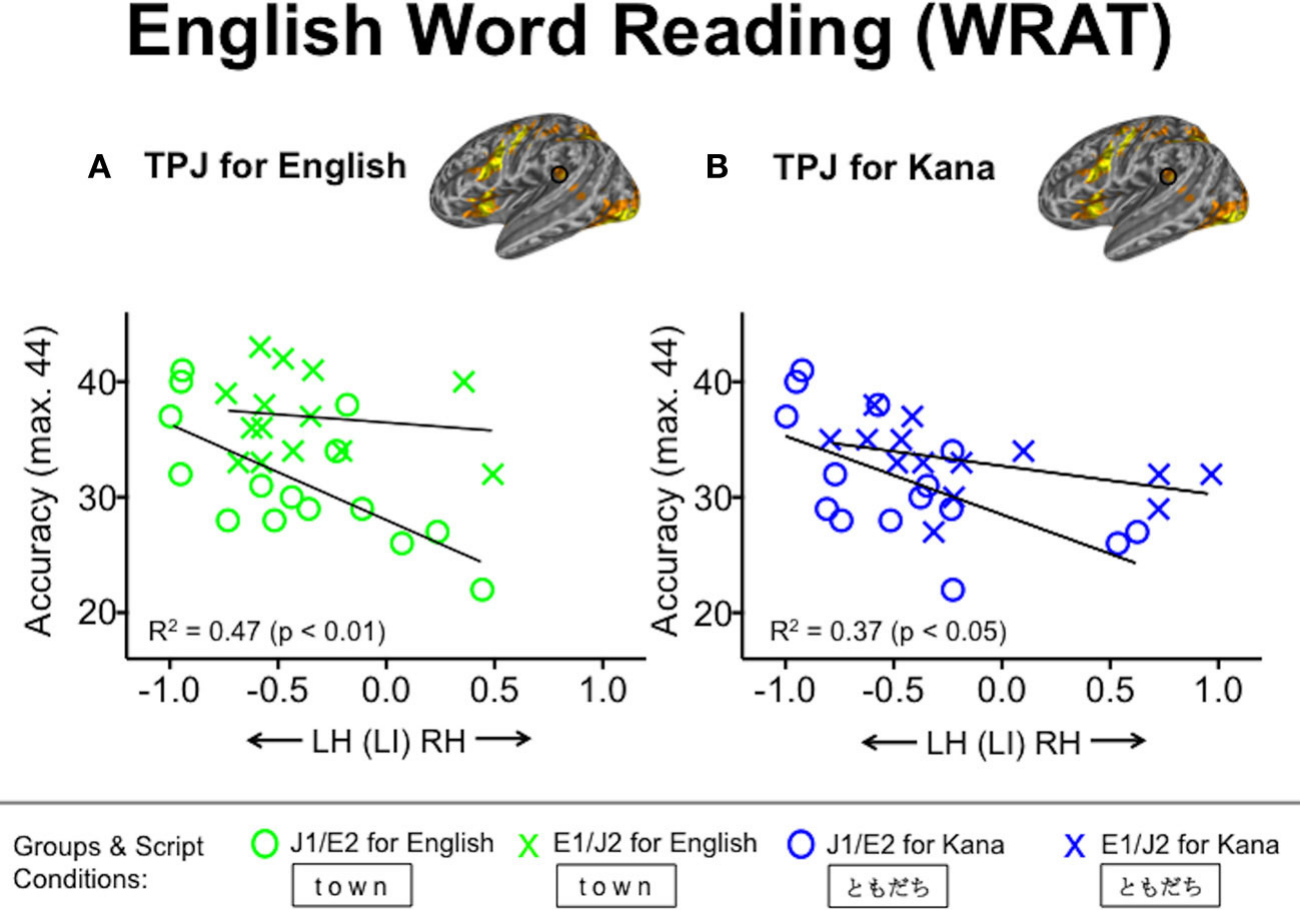
**Relationships between laterality index values in the temporo-parietal junction (TPJ) and English word reading accuracy. (A)** Green and **(B)** blue symbols represent individuals' laterality index values based on the English and Kana conditions, respectively. Accuracy was defined as the number of words pronounced correctly. WRAT, the Wide Range Achievement Test; J1/E2, Japanese L1/English L2 group; E1/J2, English L1/Japanese L2 group.

**Figure 6:**
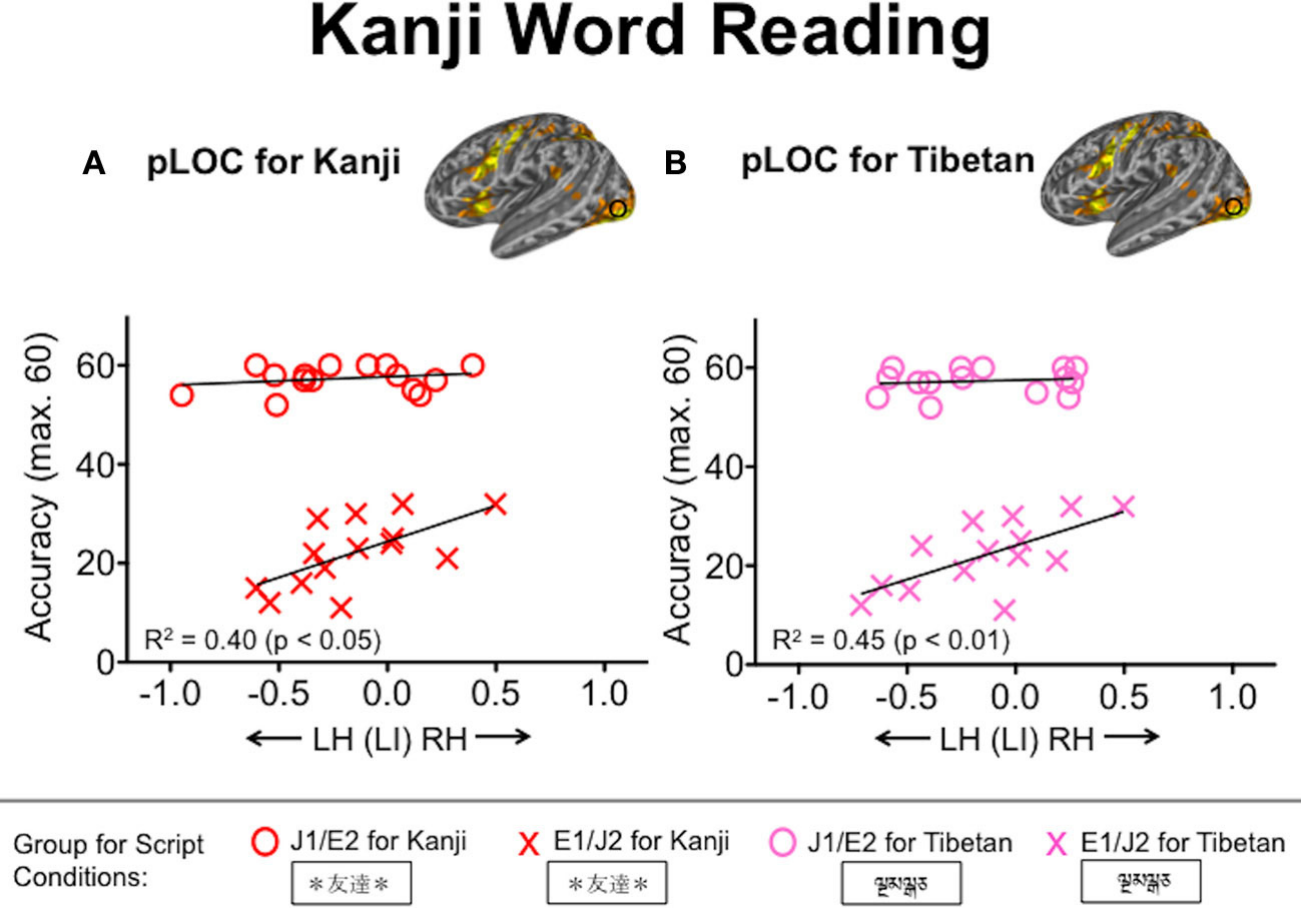
**Relationships between laterality index values in the posterior lateral occipital complex (pLOC) and Kanji word reading accuracy. (A)** Red and **(B)** pink symbols represent individuals' laterality index values based on the Kanji and Tibetan conditions, respectively. Accuracy was defined as the number of words pronounced correctly. J1/E2, Japanese L1/English L2 group; E1/J2, English L1/Japanese L2 group.

We examined the extent to which L2 word reading competency correlates with the degree of functional lateralization for visual word processing. For L2 English reading performance in the J1/E2 group, the WRAT accuracy correlated inversely with the laterality index values in the TPJ during both L2 English (*R*^2^ = 0.47, *p* < 0.01) and L1 Kana (*R*^2^ = 0.37, *p* < 0.05) conditions (Figure 5). That is, L2 readers of English who had better reading performance tended to exhibit stronger leftward functional lateralization in the TPJ not only when processing English words but also when processing Kana words. This correlation was not seen in the L1 English group. No other ROIs other than the TPJ showed significant relationships with the WRAT accuracy scores. For L2 Kanji reading performance, accuracy on the Kanji Word Reading Test correlated positively with the laterality index values in pLOC during both L2 Kanji (*R*^2^ = 0.40, *p* < 0.05) and Tibetan (*R*^2^ = 0.45, *p* < 0.01) conditions (Figure 6). That is, L2 readers of Japanese who had better reading performance in L2 Kanji tended to exhibit stronger rightward functional lateralization in pLOC not only while processing Kanji but also while processing Tibetan. Like the results for the TPJ, the laterality index in the pLOC showed no significant relationship with Kanji reading scores in the Japanese L1 group. No ROIs other than the pLOC showed significant relationships with the Kanji reading accuracy scores. Neither accuracy nor reaction time for the Kana Word Reading Test was significantly associated with laterality index values in any ROI.

## Discussion

In this study, we investigated the functional lateralization for visual word processing in two types of L2 groups, primarily focusing on the effects of visual complexity and orthographic depth on the laterality index for L2 reading. Consistent with our prediction, irrespective of whether it was participants' L1 or L2, visually complex logographic Kanji was associated with weaker leftward lateralization in the pLOC, relative to both alphabetic English and syllabic Kana conditions. This finding indicates that weaker leftward lateralization in pLOC results from the greater visuo-spatial processing demands of visually complex logographic symbols that require more right hemisphere processing (e.g., Lycke et al., 2008; Gotts et al., 2013), rather than specifically reflecting the L2 readers' extra efforts required to learn the new script. However, contrary to our prediction, there was no effect of orthographic depth on functional lateralization for L2 reading in late L2 readers. That is, no stronger leftward lateralization was observed for L2 English than for L1 Kana in the J1/E2 group. With regard to brain-behavior relationships, better L2 reading performance in English was associated with stronger leftward lateralization in the TPJ, a core region for phonological processing (e.g., Jobard et al., 2003), during both L2 English and L1 Kana conditions. For logographic L2 reading, better performance in Kanji was associated with weaker leftward lateralization in the pLOC during both the L2 Kanji and the control Tibetan conditions.

### Weaker leftward lateralization in pLOC for logographic Kanji

In the group for whom Japanese was the L2 (E1/J2), the degree of leftward lateralization in the pLOC was weaker for L2 Kanji relative to L1 English. However, this weaker leftward lateralization for Kanji in the pLOC does not specifically indicate L2 readers' extra efforts, because the native Japanese readers (J1/E2) also exhibited weaker leftward lateralization for L1 Kanji relative not only to L1 Kana but also to L2 English. This region is responsive specifically to higher-level letter shape information (Kourtzi and Kanwisher, 2001) and to visual complexity (Xu and Chun, 2006), rather than to lower-level features (e.g., edges) during visual object recognition. Hence, processing Kanji may require more global/holistic visuo-spatial processing due to its greater level of visual complexity, for which the right hemisphere is more specialized. Consequently, the right pLOC is additionally activated during Kanji reading, which contributes to the reduction of leftward lateralization in the pLOC for Kanji, irrespective of whether the script was the L1 or L2. Support for this suggestion comes from a recent fMRI study, which showed that logographic training in artificial symbols (mapping of logographic-like symbols to corresponding sounds) reduced the degree of leftward lateralization in the pLOC (posterior fusiform gyrus in their terminology) (Mei et al., 2013).

Although weaker leftward lateralization for logographic Kanji relative to other pronounceable phonographic scripts was observed in both L1 and L2 readers of Japanese, its relevance to reading competency differed between the two groups. L2 readers of Japanese (E1/J2) who exhibited weaker leftward lateralization in pLOC while processing Kanji or Tibetan tended to have better reading performance in Kanji (i.e., the E1/J2 readers with more rightward lateralization had the highest Kanji reading scores). However, this relationship was not seen in the L1 group of Japanese (J1/E2). Recently, Gotts et al. (2013) have demonstrated a dissociation in the specialized functions of the left and right hemispheres, highlighting that rightward lateralization predicts better visuo-spatial attentional performance. As the Kanji and Tibetan conditions were more visually demanding (the Kanji symbols are visually complex and the Tibetan letter strings could only be processed visually), the brain-behavior relationships in the E1/J2 group indicate that strong rightward lateralization, rather than strong leftward lateralization, may be beneficial for visual processing of logographic words in late L2 readers of Japanese. In other words, reliance on right hemisphere functions may allow for the successful recognition of the visually demanding logographic symbols in less proficient L2 readers of logographic scripts.

Consistent with previous studies (Nakamura et al., 2005b; Seghier and Price, 2011), leftward lateralization was found for both pronounceable and unpronounceable scripts. As suggested by Seghier and Price (2011), acquiring reading expertise can reduce the involvement of the right hemisphere, which can result in the increased degree of leftward lateralization in pLOC for learnt symbols/letters. This is supported by our finding showing stronger leftward lateralization for pronounceable conditions relative to unpronounceable conditions (e.g., the Tibetan for the J1/E2 and E1/J2 groups). That said, the effect of reading expertise on leftward lateralization in the pLOC may be limited to visually demanding logographic symbols, even in L1 logographic readers, as was clearly shown in the weaker leftward lateralization for Kanji than Kana in the Japanese L1 group.

### No stronger leftward lateralization for l2 alphabetic English

Contrary to our prediction, there was no stronger leftward lateralization observed for L2 English reading in the J1/E2 group, indicating a limited effect of orthographic depth on functional lateralization for L2 reading. However, considering our previous findings showing greater activation in a phonological region in the left hemisphere for L2 English reading relative to L1 Kana reading during a phonological task (Koyama et al., 2013), it remains possible that the visual task used may not have been sensitive enough to assess L2 readers' extra efforts to cope with the greater phonological demands of L2 English reading. Mei et al. (2013) demonstrated that phonological training (phonetic mapping) using artificial symbols increased leftward lateralization in the posterior fusiform gyrus (adjacent to pLOC in the current study). This effect of phonetic learning/experience was clearly reflected in the pattern of functional lateralization in the control group: there was stronger leftward lateralization in the majority of ROIs for the English condition relative to the other unpronounceable/unfamiliar conditions. Therefore, it is possible that increased leftward lateralization associated with increased phonological demands for L2 reading might be observed during a phonological task.

Although no difference was observed between the L1 and L2 phonographic scripts, functional lateralization in the TPJ was relevant to reading competency only in the L2 readers of English (J1/E2 group). More specifically, higher L2 reading scores in English were associated with stronger leftward lateralization in the TPJ, which has been shown to be crucial for phonological decoding (e.g., Welcome and Joanisse, 2012), during not only the L2 English condition but also the L1 Kana condition. This result indicates that functional lateralization for written L1 words can predict word reading competency in L2 when both L1 and L2 are phonographic scripts. This is consistent with previous behavioral findings that phonological and reading skills in an alphabetic L1 can predict later L2 reading in another alphabetic script (Sparks et al., 2006, 2012). Of note, this cross-script transfer of reading competency has been observed between L1 logographic and L2 alphabetic reading at the behavioral level (Chuang, 2011), but was absent in the current fMRI study.

In conclusion, the current study provides evidence that weaker leftward lateralization is associated with greater involvement of right hemisphere visuo-spatial processing, rather than specifically reflecting L2 readers' additional efforts. Visually complex logographic symbols rely more on the functions of the right hemisphere; particularly those of the right posterior lateral occipital complex (pLOC), relative to phonographic symbols (alphabetic and syllabic), even after extensive reading experience (evident in L1 readers). For late L2 readers of logographic scripts (Japanese Kanji and probably Chinese), strong rightward lateralization (rather than strong leftward lateralization) in pLOC may be beneficial for L2 word reading, at least for visual word recognition. In contrast, when L1 and L2 are both phonographic scripts where symbols are mapped onto sounds, stronger leftward lateralization in the temporo-parietal junction (a region crucial for phonological processing) during L1 word processing can predict better L2 reading competency. Further research is necessary to investigate functional lateralization in a larger sample during reading tasks that selectively tap either phonological or semantic components, as well as its relevance to L2 reading performance.

## Supplementary material

The Supplementary Material for this article can be found online at: http:www.frontiersin.org/journal/10.3389/fnhum.2014.00249/abstract

Supplementary Figure 1 **(A)** Functional lateralization in the fusiform gyrus (FFG) for each script condition, and **(B)** group comparisons of functional lateralization for the Tibetan condition in the FFG. Vertical error bars on data points represent the standard error of the mean. LH, Left Hemisphere; RH, Right Hemisphere; LI, Laterality Index; J1/E2, Japanese L1/English L2 group; E1/J2, English L1/Japanese L2 group; Control, Monolingual English L1 control group. ^**^p < 0.01, ^*^p < 0.05.

Supplementary Figure 2 **The BOLD signal changes in the left and right pLOC for each script condition relative to the resting condition in the J1/E2 group**. LH, Left Hemisphere; RH, Right Hemisphere. ^*^p < 0.01.

### Conflict of interest statement

The authors declare that the research was conducted in the absence of any commercial or financial relationships that could be construed as a potential conflict of interest.
