# Supplementary figures and images for: A cross-linguistic evaluation of script-specific effects on fMRI lateralization in late second language readers
Source: Front Hum Neurosci. 2014 Apr 24;8:249. doi: 10.3389/fnhum.2014.00249 (PMC4006067; doi:10.3389/fnhum.2014.00249)

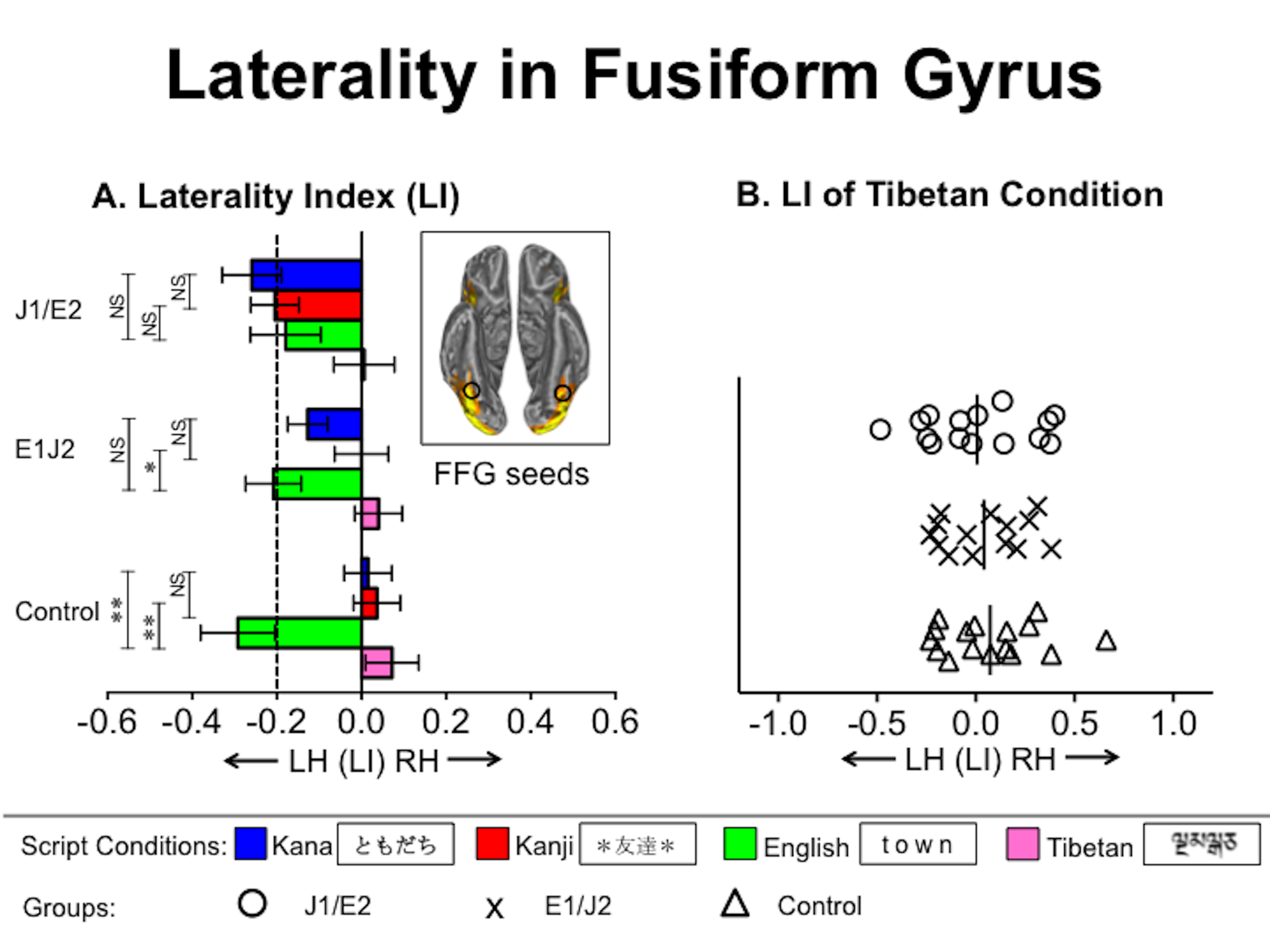

Supplement: Supplementary Figure 1 — (A) Functional lateralization in the fusiform gyrus (FFG) for each script condition, and (B) group comparisons of functional lateralization for the Tibetan condition in the FFG. Vertical error bars on data points represent the standard error of the mean. LH, Left Hemisphere; RH, Right Hemisphere; LI, Laterality Index; J1/E2, Japanese L1/English L2 group; E1/J2, English L1/Japanese L2 group; Control, Monolingual English L1 control group. **p < 0.01, *p < 0.05. [file Presentation1.ZIP › 76272_Koyama_Suppl_Figure_1.TIF]

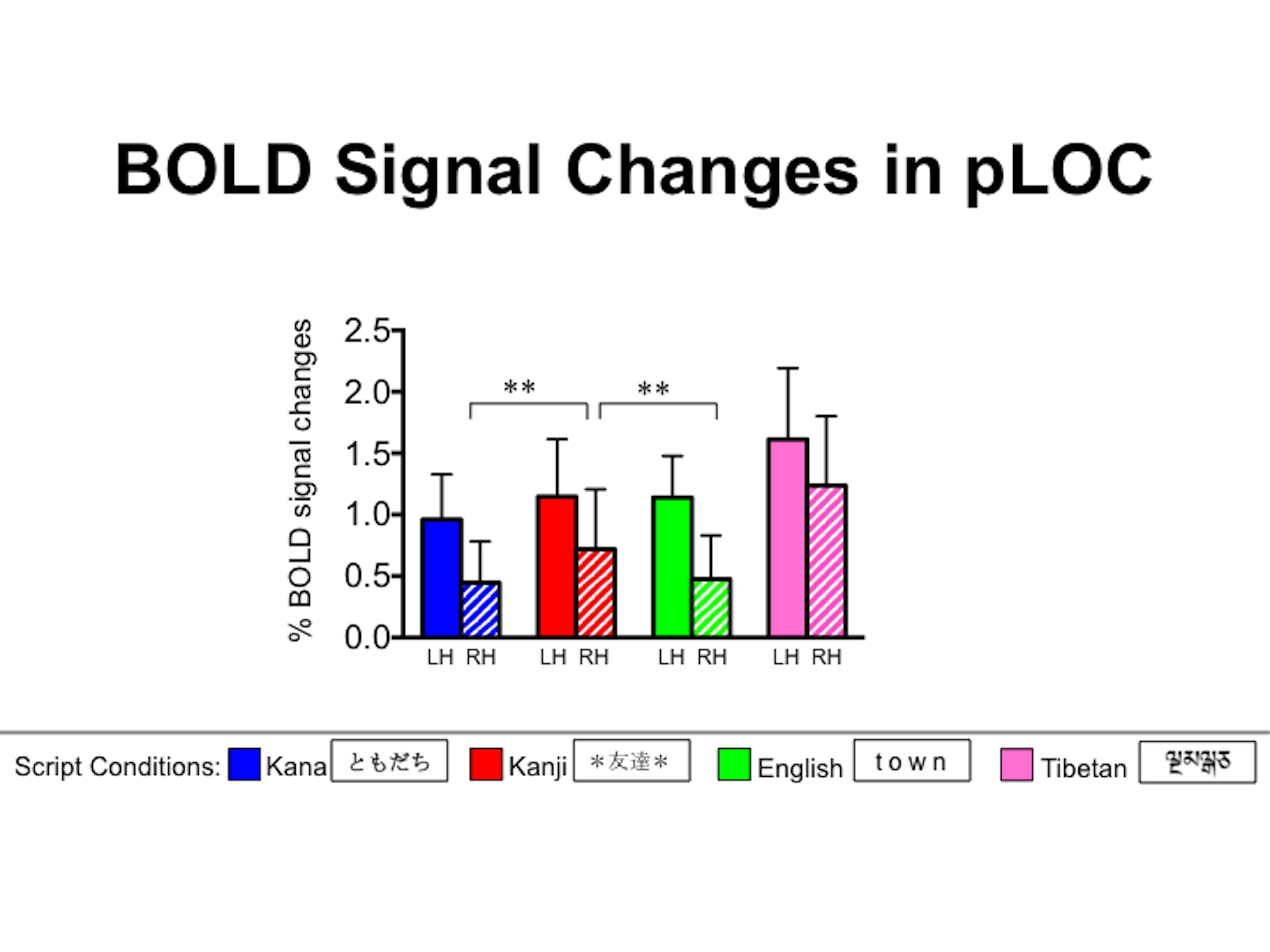

Supplement: Supplementary Figure 1 — (A) Functional lateralization in the fusiform gyrus (FFG) for each script condition, and (B) group comparisons of functional lateralization for the Tibetan condition in the FFG. Vertical error bars on data points represent the standard error of the mean. LH, Left Hemisphere; RH, Right Hemisphere; LI, Laterality Index; J1/E2, Japanese L1/English L2 group; E1/J2, English L1/Japanese L2 group; Control, Monolingual English L1 control group. **p < 0.01, *p < 0.05. [file Presentation1.ZIP › 76272_Koyama_Suppl_Figure_2.TIF]
